# Do Atopic Dermatitis and Psoriasis Have an Impact on Cognitive Decline—Latest Research Review

**DOI:** 10.3390/healthcare12121170

**Published:** 2024-06-10

**Authors:** Marcin Kuryłło, Ewa Mojs

**Affiliations:** Department of Clinical Psychology, Poznan University of Medical Sciences, 60-812 Poznań, Poland; ewa_mojs@poczta.onet.pl

**Keywords:** atopic dermatitis, psoriasis, cognitive impairment, cognitive processes

## Abstract

Background: Atopic dermatitis and psoriasis are chronic skin diseases that affect the mental health of patients. The relationship between AD and psoriasis and cognitive processes in patients remains unclear. The aim of the review was to answer the question of whether AD and psoriasis have an impact on cognitive decline in patients. Method: A systematic literature search was conducted on PubMed and EBSCO to identify case–control, cross-sectional, or cohort studies that evaluated the association between atopic dermatitis and psoriasis and cognitive impairment. Results: Most of the studies included in the review confirmed cognitive decline in patients with atopic dermatitis and psoriasis. Conclusions: It seems that atopic dermatitis and psoriasis may negatively affect cognitive processes such as working memory, concentration, attention, and speed of motor reactions. Psychological interventions targeting distorted cognitive processing could improve the quality of life of patients with atopic dermatitis and psoriasis.

## 1. Introduction

Atopic dermatitis and psoriasis are common, chronic skin diseases affecting men and women all over the world [1,2]. AD is estimated to affect 15–30% of children and 2–10% of adults [3,4,5]. AD is characterized by impaired epidermal barrier function, sensitive and dry skin, eczematous lesions that can be localized or disseminated, and intense itching [6]. The prevalence of psoriasis in respective countries ranges from 0.09% to 11.43% [7]. Lesions typically appear as well-defined, erythematous, itchy papules and plaques covered with dry, white, or silvery scales, commonly on the skin of the elbows, knees, lumbosacral region, and scalp. However, they can appear anywhere on the body [6].

Both diseases are associated with an increased risk of comorbidities. Atopic dermatitis may increase the risk of other atopic disorders, including asthma, hay fever, food allergies and eosinophilic oesophagitis [8]. Whereas patients with severe psoriasis face an increased risk of death due to malignant neoplasms, chronic lower respiratory tract infections, diabetes and kidney diseases [9]. Both groups are more susceptible to the risk of dementia, infections and cardiovascular diseases [8,9].

In recent years, there have been many studies on the correlation between psoriasis and AD and mental condition of patients [10,11,12,13]. Numerous publications indicate the high prevalence of anxiety and depressive symptoms [14,15], suicidal thoughts [16,17,18,19] and alcohol abuse [20,21] among patients with AD and psoriasis.

The association between AD and psoriasis and cognitive processes in patients is still unclear. Cognitive impairment is demonstrated through memory difficulties [22], and difficulties with concentration and decision-making, which translates to the quality of life of the patients. The symptoms range from mild to acute, and the prevalence of cognitive impairment differs depending on the age, level of education and geographical location [23]. Cao S. et al. (2024) emphasized the significant causal association between AD and an increased risk of autism spectrum disorder and also identified bipolar disorder and anorexia nervosa as risk factors for atopic dermatitis [24]. Earlier reports have demonstrated a relationship between AD and psoriasis, and cognitive functions [25,26,27]. However, the studies were scarce in number. It was only recently, that the interest in this issue has grown, and more publications with study-based findings were published.

## 2. Method

**Figure 1:**
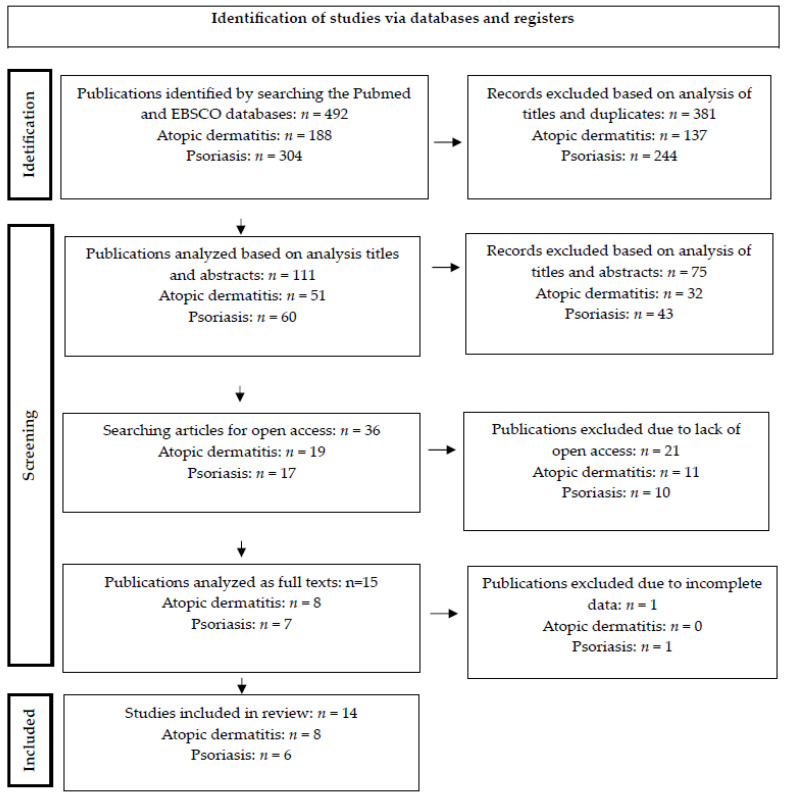
Flow chart of the review process according to PRISMA guidelines.

The review was conducted in accordance with the PRISMA 2020 Statement (Preferred Reporting Items for Systematic Reviews and Meta-Analyses). The PRISMA statement and its extensions are evidence-based recommendations intended to encourage transparent and comprehensive reporting of systematic reviews. The 2020 PRISMA statement replaced the 2009 statement, which was originally developed to help systematic reviewers be transparent about why a review was conducted, what the authors executed, and what they found. PRISMA 2020 includes new reporting guidelines that reflect advances in methods for identifying, selecting, evaluating and synthesizing studies [28] (see Figure 1).

A systematic review of the scientific literature in PubMed and EBSCO [eBook Open Access (OA) Collection, Academic Search Ultimate, APA PsycInfo, APA PsycArticles, eBook Collection, APA PsycBooks, ERIC, Health Source: Nursing/Academic Edition, Library, Information Science & Technology Abstracts, MEDLINE, Academic Research Source eJournals)] services was performed to identify case–control, cross-sectional or cohort studies where the relationship between atopic dermatitis and psoriasis, and cognitive disorders was assessed. The authors of the review wanted to find an answer to the question whether AD and psoriasis affect the impairment of cognitive functions in patients.

The methodology of the review was formally discussed and agreed within the study group. While searching for the studies, the articles published within the last 6 years (from January 2018 to 18 March 2024) were considered. The searched entries included both the standard headings of medical topics and keywords, such as atopic dermatitis, AD, eczema, psoriasis, plaque psoriasis, cognitive functions, cognitive processes, dementia, memory, attention, thinking, executive functions. The entries were mixed with Boolean operators, and the searched entries were combined and thus it was possible to develop several potential search strategies to find the appropriate results. The included criteria are presented in Table 1.

Only those studies that included control groups were presented. The review includes articles whose full content is available in open access. 

Exclusion criteria: meta-analyses, reviews, systematic reviews, scoping reviews, conference reports, commentaries, theses and non-peer-reviewed publications were excluded from the review. 

A computer search for articles was performed in accordance with the identified criteria. First, duplicates were removed from 492 records; then, authors studied the titles. Next, after analyzing the titles and abstracts, the authors excluded 75 articles because they did not comply with the criteria established by the authors. The authors identified 36 potentially relevant articles that clearly presented the premises and research objectives, explaining the purposefulness of sample selection and participant recruitment. Attention was paid to whether appropriate research tools were used, the transparency of research financing sources and open access. The next step was to select open-access articles. The full texts of the selected articles were fully analyzed to avoid the risk of missing potentially important data. Finally, the authors decided to include 14 studies in the presented results. All studies included a control group. The results are presented in Table 2 and Table 3:

Due to the fact that the primary studies included in the review were heterogeneous and there were differences in the study groups, methods and measurement tools used, it is impossible to conduct a meta-analysis, so the authors decided to use a narrative approach.

## 3. Results

### 3.1. Effects of Atopic Dermatitis on Cognition

Three of the eight studies focused on children. Two of the three studies [30,32] indicated memory disorders, developmental delays and neurodevelopmental problems, including those related to attention and hyperactivity, in children with AD. One other study [31] found no greater cognitive problems in children with atopic dermatitis than in healthy children.

Two of the eight studies [33,35] focused on dementia, and both confirmed a greater risk of developing dementia in people with atopic dermatitis compared to healthy people. The first study [33] showed that the association increased with the severity of atopic eczema. The authors of the second study [35] showed a significant burden of dementia in patients with atopic dermatitis.

One of the eight studies [29] found no significant difference between the atopic dermatitis group and the healthy group in terms of tasks related to cognitive abilities. However, it was shown that people with atopic dermatitis had higher scores on the depression and stress scale.

One of the eight studies [34] did not confirm increased cognitive problems in young men with AD. Swedish males with atopic dermatitis had lower stress tolerance in late adolescence but did not have poorer cognitive function or educational achievement.

One of the eight studies [6] indicated a shared prevalence of right-hemispheric dominance in both atopic dermatitis and psoriasis patients. AD patients with dominant right hemisphere prefrontal activation showed increased markers of inhibition and avoidance, while psoriasis patients showed increased sympathetic nervous system activity.

### 3.2. Effects of Psoriasis on Cognition

Three of the six studies [36,39,40] confirmed the problems of psoriasis patients associated with worse results in neuropsychological tests and showed more intense changes in MRI compared to the healthy control group and greater problems with concentration and attention.

Three of the six studies [37,38,41] focused on dementia and Alzheimer’s disease. Two of three studies [37,41] confirmed a greater risk of developing dementia in people with psoriasis compared to healthy people. One study [38] showed that people with psoriasis have a lower incidence of dementia.

One of the studies included in Table 2 [6] indicated a shared prevalence of right-hemispheric dominance in psoriasis patients.

## 4. Discussion

Despite advances in understanding the pathophysiology and introducing new treatments for chronic skin conditions, including atopic dermatitis and psoriasis, surviving and treating the disease place a heavy burden on patients, their families and society. The aim of the paper was to present the latest research on the impact of atopic dermatitis and psoriasis on cognitive processes in patients.

Many of the studies published so far have shown that the prevalence of cognitive impairment is higher in people with certain inflammatory skin diseases, including psoriasis and chronic eczema dermatitis [22,23,42,43]. Nine studies included in this review are coherent with those findings [30,32,33,35,36,37,39,40,41].

Yi et al. (2024) assessed alterations of intrinsic brain activity and its association with cognitive function in patients with psoriasis. As reported, patients with psoriasis had altered brain activity and connectivity in the key brain areas within the DMN-prefrontal circuit. According to the authors, these brain changes may be the underlying neural correlates for cognitive functioning in patients with psoriasis [44]. The review included a study on the association between the variation in the function of the right and left hemispheres of the brain and the prevalence of atopic dermatitis and psoriasis. Frontal asymmetry is often treated as a predictor or consequence variable regarding motivation, emotional regulation, and psychopathology [6,45]. Each hemisphere of the brain plays a specialized role in cognitive and behavioural processes; therefore, in chronic skin diseases such as psoriasis and atopic dermatitis, the degree of lateralization between the hemispheres may provide insight into the specific links between skin diseases and the psyche. The authors presented data that suggest the dominance of the right hemisphere in both AD and psoriasis patient groups. The study also examined the cognitive and metacognitive abilities of the subjects, but no significant differences were found in these areas between the patients and the control group [6].

Research on the impact of chronic skin diseases in children, including atopic dermatitis and psoriasis, on cognitive processes is inconsistent. Lee et al. (2023) showed that AD was associated with vulnerability in concentrations (aOR: 1.170; CI: 1.093–1.254). The impact of AD on concentrations showed consistent results regardless of sex, exposure to systemic corticosteroids and antihistamines, and age at the diagnosis of AD [46]. A study by Ma EZ (2024) suggested that pediatric AD was generally associated with greater odds of reported difficulties in learning and memory. However, this association was primarily limited to children with neurodevelopmental comorbidities, such as ADHD or learning disabilities [47]. Vittrup et al. (2023) reported that atopic dermatitis, particularly when severe, is associated with lower school performance in childhood and IQ in young men, which can interfere with academic achievements in life [48]. Sockler et al. (2024) did not find any clinically meaningful associations between AD activity and severity and general cognitive function during early childhood and adolescence [49]. The studies included in this review confirm these inconsistencies. Two of the four studies [30,32] indicate greater neurodevelopmental problems in children with AD. One other study [31] found no greater cognitive problems in children with atopic dermatitis than in healthy children.

Similar to studies on the impact of chronic skin diseases on cognitive processes in children, studies on the impact of atopic dermatitis and psoriasis on dementia and Alzheimer’s disease are also inconclusive. Two separate teams of researchers Wu CY et al. (2020) and Pezzolo et al. (2021) reported that psoriasis was not found to be a risk factor for dementia [50] and was not associated with preclinical markers or higher dementia risk [51]. While other researchers (Huang KL. et al., 2019) reported a significantly higher risk of dementia (aHR = 1.23; 95% CI: 1.06–1.42) identified in the psoriasis group [52]. Also, Khalaf et al. (2021) confirmed worse cognitive impairment when compared to the controls regardless of the psoriasis severity [53]. Joh et al. (2023) showed that asthma, allergic rhinitis, and atopic dermatitis were significantly associated with increased risk of all-cause dementia and subtypes, with dose-effect relationships with the severity of allergic diseases [54]. Some of the presented research results attempt to answer the question of whether atopic dermatitis and psoriasis influence dementia and Alzheimer’s disease [33,35,37,38,41]. The risk of dementia includes genetic factors, aging, environmental factors, certain types of diseases, and unhealthy lifestyles. Psoriasis usually occurs much earlier than dementia; therefore, according to some researchers, there is reason to speculate that psoriasis may also be a risk factor for dementia [55]. A large cross-sectional study from Israel showed an inverse relationship between psoriasis and dementia, patients with psoriasis were less likely to develop dementia than control groups [38]. 

This review has some limitations. First, only a small number of the interventions presented in this review included randomised controlled trials. Second, although the methodological aspects of experimental design were duly taken into account in the interventions presented, the observations collected here differed in terms of sample size, experimental procedure, approaches, and outcome measures. 

Both atopic dermatitis and psoriasis cause a lot of suffering to patients, which is often associated with stress and, as a consequence, mental problems such as depression or anxiety [14,15,56]. Many psychological interventions have been tested for atopic dermatitis and psoriasis [6,57,58,59,60] but personalized approaches are lacking due to the potential prevalence of cognitive impairment. Due to the fact that most of the findings of the presented studies indicate the existence of the impact of AD and psoriasis on cognitive functions, it is worth considering the inclusion of psychological interventions in medical treatment conducted by dermatologists. Jen H. et al. (2021) suggested brief cognitive assessments to screen older psoriasis patients with subjective cognitive complaints [61]. Pan TL. et al. (2021) indicated that atopic dermatitis may be an independent risk factor for new-onset dementia and clinicians may monitor the trajectory of neurocognitive function among elderly patients with AD [62].

Overall, this review contributes to the existing literature on the effects of AD and psoriasis on cognition and highlights the importance of further research on this topic.

## 5. Conclusions

1. Most available studies indicate a negative impact of chronic skin diseases, including atopic dermatitis and psoriasis, on cognitive function in patients;
2. Further research is needed to examine the impact of chronic skin diseases, including atopic dermatitis and psoriasis, on cognitive impairment;
3. The use of psychological interventions targeting distorted cognitive processing in patients with atopic dermatitis and psoriasis could improve their quality of life.

## Figures and Tables

**Table 1:** Included criteria according to PECOS.

Population (P)	Exposure (E)	Comparison (C)	Outcome (O)	Study Design (S)
People suffering from psoriasis or atopic dermatitis.	Neuropsychological tests, neuroimaging.	Comparison with a group of healthy people.	Impact on cognitive processes.	Control, prospective, retrospective, longitudinal, observational, cohort or cross-sectional studies.

Source: own study.

**Table 2:** Effects of atopic dermatitis on cognition.

	First Author and Year	Study Design	Aim/Domains Assessed	Study Group	Conclusions
1	Bozsányi S, et al., 2023 [6]	Observational study	Assessment of Frontal Hemispherical Lateralization in Plaque Psoriasis and Atopic Dermatitis.	*N* = 46 patients with psoriasis, *N* = 56 patients with AD, *N* = 29 people without skin diseases, Hungary.	Psychophysiological and psychometric data suggest a shared prevalence of right-hemispheric dominance in both AD and Pso patient groups.
2	Fereidouni M, et al., 2021 [29]	Cross-sectional study	A study of the association of cognitive abilities and emotional function with allergic disorders in young women.	*N* = 181 female students (*N* = 54 Allergic Rhino-conjunctivitis, Eczema, Asthma, *N* = 127 healthy women), between 18 and 27 years of age in Iran.	There was a high prevalence of psychological/psychiatric disorders that included: anxiety, and sleep problems among allergic women. Those with at least one allergy disorder were more likely to have anxiety behavior than healthy individuals (Odds ratio = 1.86; 95% CI: 1.02–3.4), and insomnia symptoms (OR = 2.3; 95% CI: 1.2–4.3).
3	Kim JH, et al., 2023 [30]	Cross-sectional study	Neurodevelopment at 6 years of age in children with atopic dermatitis.	*N* = 30,557 children with AD, *N* = 89,452 healthy children, born between 2008 and 2012 in Korea.	AD before age 2 years may be associated with an increased risk of neurodevelopmental dysfunction including gross and fine motor skills in the young childhood period. AD group showed a higher risk of suspected neurodevelopmental dysfunction in the total score (weighted adjusted odds ratio: 1.10; 95% CI: 1.05–1.16], gross motor skills: 1.14; CI 1.04–1.25, and fine motor skills: 1.15; CI 1.06–1.25) than the control group.
4	Kuo HC, et al., 2020 [31]	Observational study	Allergic diseases do not impair the cognitive development of children but do damage the mental health of their caregivers.	*N* = 109 patients with AD (mean age 6.8 years) *N* = 82 healthy children (mean age 6.3 years) from Taiwan.	Allergic diseases did not impair the cognitive development of children. Atopic dermatitis did not exhibit an individual effect on children’s cognitive scores, ADHD symptoms and severity (SNAP-IV) scores, or caregivers’ family APGAR scores.
5	Jackson-Cowan L, et al., 2018 [32]	Cross-sectional study	Childhood atopic dermatitis is associated with cognitive dysfunction: A National Health Interview Survey study from 2008 to 2018.	*N* = 13,398 children with AD, *N* = 96,084 children without AD, at ages 2–17 years, USA.	The prevalences of cognitive dysfunction, such as memory impairment (0.87% vs. 0.42%), developmental delays (6.96% vs. 3.87%), and attention deficit (hyperactivity) disorder (10.78% vs. 8.10%) were higher in children with vs. without AD.
6	Magyari A, et al., 2022 [33]	Longitudinal cohort study	Adult atopic eczema and the risk of dementia	*N* = 213,444 patients diagnosed with Atopic eczema, *N* = 1554 223 healthy individuals, aged 60 to 99 years, UK.	Atopic eczema was associated with a small but increased risk of incident dementia. The association increased with the severity of atopic eczema. Participants with atopic eczema had a 27% increased risk of dementia after adjusting for the potential confounders (hazard ratio (HR): 1.27; 95% CI: 1.23–1.30). The magnitude of risk increased with increasing atopic eczema severity.
7	Smirnova J, et al., 2019 [34]	National cohort study	Atopic dermatitis, educational attainment and psychological functioning: a national cohort study.	*N* = 1673 males with AD, *N* = 233,042 males without AD at ages 17–20 years assessed for military conscription in Sweden between 1969 and 1976.	Swedish men with AD did not have lower cognitive function or poorer educational attainment. AD was associated with a greater risk of low stress resilience (adjusted relative risk ratio (RRR) 1.60; 95% confidence interval 1.38 to 1.86). AD was associated with higher cognitive function (b coefficient 0.15; 0.05 to 0.24) and higher educational level (RRR 1.29; 1.13 to 1.47) but adjustment for socioeconomic characteristics of the family of origin attenuated the magnitude of the associations and eliminated statistical significance (b coefficient 0.06; −0.03 to 0.15) and (RRR 1.16; 1.00 to 1.35).
8	Woo YR, et al., 2023 [35]	National cohort study	Increased risk of Dementia in patients with atopic dermatitis.	*N* = 38,391 people with AD, *N* = 2,643,602 people without AD adults ≥ 40 years of age, Korean National Health Insurance System (NHIS) database from 2009 to 2016.	The risks of all-cause dementia and Alzheimer’s disease were increased in patients with AD. Males with AD had an increased risk of dementia (HR: 1.111; 95% CI: 1.040–1.186) and Alzheimer’s disease (HR: 1.099; 95% CI: 1.019–1.184) compared with males without AD.

Source: Own study.

**Table 3:** Effects of psoriasis on cognition.

	First Author and Year	Study Design	Aim/Domains Assessed	Study Group	Conclusions
1	Deveci E, et al., 2019 [36]	Observational study	Oxidative stress and inflammatory response in patients with psoriasis; is there any relationship between psychiatric comorbidity and cognitive functions?	*N* = 37 patients diagnosed with psoriasis, *N* = 37 healthy volunteers, aged between 18 and 65 years, Turkey.	Psoriasis patients have higher risk factors than healthy controls for cognitive impairment, independent of depression, inflammation and oxidative stress levels. The control group’s Öktem Learning (*t*-test = 3.756, *p* < 0.001); Phonemic Verbal Fluency Test (K-A-S), KAS-K (t = 3.615, *p* < 0.001), KAS-A (t = 3.391, *p* < 0.001), KAS-S (t = 4.441, *p* < 0.0001), scores and completed category number were higher than the patient group (t = 2.082, *p* = 0.041) according to Wisconsin Card Sorting Test. Total recall scores of the control group were higher than patients (U = 503.00, *p* < 0.05) according to Mann–Whitney U test.
2	Kim M, et al., 2020 [37]	National cohort study	Increased risk of Alzheimer’s disease in patients with psoriasis	*N* = 535,927 patients diagnosed with psoriasis, *N* = 2,679,635 healthy subjects, aged 40–64 years, Korea.	In a multivariable-adjusted model, patients with psoriasis showed a significantly increased risk of Alzheimer’s disease (hazard ratio: 1.09; 95% CI: 1.07–1.12, *p* < 0.0001) compared to controls without psoriasis. Among patients with psoriasis, the risk of Alzheimer’s disease was significantly increased in psoriasis patients not receiving systemic therapy compared to those receiving systemic therapy (hazard ratio: 1.10; 95% CI, 1.08–1.12 vs. hazard ratio: 0.99; 95% CI: 0.90–1.09, *p* < 0.0001).
3	Kridin K, et al., 2020 [38]	Cross-sectional study	Psoriasis and Dementia	*N* = 121,801 patients with psoriasis, *N* = 121,802 healthy subjects from Israel, the mean age = 48.9 years.	Psoriasis was associated with a lower prevalence of dementia relative to control subjects. Multivariate analysis adjusting for demographic variables, cardiovascular-related risk factors, and healthcare utilization demonstrated a significant inverse association between psoriasis and dementia in the entire study population (adjusted OR 0.86; 95% CI 0.76–0.96; *p* = 0.009), but not in the subgroup of patients with moderate-to-severe psoriasis (adjusted OR 0.91; 95% CI 0.81–1.02; *p* = 0.113).
4	Marek-Józefowicz L, et al., 2022 [39]	Observational study	Cognitive functions associated with brain imaging markers in patients with psoriasis	*N* = 53 patients with psoriasis, *N* = 36 healthy controls, aged 21–68 years, Poland.	Patients with psoriasis presented worse achievements on all the neuropsychological tests and showed more intense changes on MRI compared to healthy controls. The severity of psoriasis was positively correlated with the intensity of depressive symptoms (R = 0.46, *p* = 0.01) and the time of performance on the Trail Making Test (TMT) part A (R = 0.43, *p* = 0.02). Depressive symptoms were also correlated with performance on the TMT A (R = 0.43, *p* = 0.02).
5	Padma K, et al., 2020 [40]	Cross-sectional study	Cognitive impairment in patients with psoriasis: a clinical study in teaching hospital	*N* = 100 patients with psoriasis, *N* = 100 healthy controls, aged 20–65 years, India.	Patients with psoriasis had cognitive deficits in the domain of attention, concentration and total scores of Standard Mini-Mental Status Examination (SMMSE) and Brief Cognitive Rating Scale (BCRS) for assessing cognitive functions. Study reveals statistical significance between the duration of psoriasis (*p* = 0.007) and cognitive impairment.
6	Zingel R, et al., 2023 [41]	Longitudinal cohort study	Association between psoriasis and dementia.	*N* = 10,583 patients with psoriasis, *N* = 10,583 healthy controls, over 60 years, Germany.	The study found a positive association between psoriasis and all-cause dementia in patients in general practices in Germany. Psoriasis was significantly associated with a dementia risk (HR: 1.24; 95% CI: 1.14–1.35, *p* < 0.001).

Source: Own study.

## Data Availability

The authors confirm that the data supporting the findings of this study are available within the article.
